# Robot-assisted vs. laparoscopic-assisted surgery for choledochal cyst in children: a systematic review and meta-analysis

**DOI:** 10.3389/fped.2026.1811576

**Published:** 2026-04-28

**Authors:** Fei Liu, Ke Zhang

**Affiliations:** 1 Department of General Surgery, The Second People's Hospital, Yibin, Sichuan, China; 2 Department of Pediatric Surgery, The Second People's Hospital, Yibin, Sichuan, China

**Keywords:** children, choledochal cyst, laparoscopy, meta-analysis, robot-assisted

## Abstract

**Objective:**

Comprehensive analyses specifically comparing robot-assisted surgery(RS) and laparoscopic surgery(LS) for choledochal cyst(CC) in children remained scarce. This study aimed to evaluate the safety and efficacy of RS vs. LS for pediatric CC.

**Methods:**

A systematic search of PubMed, Embase, Web of Science, and the Cochrane Library was conducted to identify studies published up to December 31, 2025, that compared laparoscopic and robot-assisted surgery for CC in children. Meta-analysis was performed using Stata 18.

**Results:**

A total of 19 retrospective studies were included. Meta-analysis results demonstrated that the RS group had significantly lower incidences of postoperative biliary stones (OR = 0.10, 95% CI: 0.01–0.89), bile leakage (OR = 0.28, 95% CI: 0.11–0.70), anastomotic stricture (OR = 0.27, 95% CI: 0.12–0.65), and overall complications (OR = 0.26, 95% CI: 0.13–0.51) compared to the LS group. Total operative time was longer in the RS group (SMD = 1.02; 95% CI: 0.30–1.74), whereas intraoperative blood loss was significantly lower (SMD = −1.22; 95% CI: −2.19 to −0.24). Additionally, the RS group exhibited shorter hepaticojejunostomy time (SMD = −1.43; 95% CI: −2.30 to −0.56), drainage tube indwelling time (SMD = −0.74; 95% CI: −1.01 to −0.47), postoperative fasting time (SMD = −0.80; 95% CI: −1.11 to −0.50), and hospital stay (SMD = −1.16; 95% CI: −2.08 to −0.23). No significant differences were observed in other outcomes, including postoperative cholangitis (OR = 0.59, 95% CI: 0.22–1.57), residual cyst (OR = 0.22, 95% CI: 0.02–1.94), incision infection (OR = 0.17, 95% CI: 0.02–1.41), intestinal obstruction (OR = 0.97, 95% CI: 0.40–2.33), pancreatitis (OR = 0.74, 95% CI: 0.08–6.47), pancreatic leakage (OR = 0.43, 95% CI: 0.10–1.92), and conversion to open surgery (OR = 0.79, 95% CI: 0.36–1.75). Furthermore, no statistically significant difference was found in cyst excision time between the two groups (SMD = −1.77; 95% CI: −3.91 to −0.77).

**Conclusion:**

In the treatment of pediatric CC, RS offered potential advantages over LS in terms of reducing postoperative biliary-related complications, decreasing intraoperative blood loss, accelerating postoperative gastrointestinal function recovery, and shortening hospital stay. Although RS required longer operative time, this limitation might be mitigated with accumulated surgical experience and technological advancements. However, current evidence is primarily derived from retrospective studies conducted in Asian countries and lacked long-term follow-up data. Well-designed multicenter prospective studies or randomized controlled trials were urgently needed to provide higher-level evidence and further validate our findings.

## Introduction

Congenital choledochal cyst (CC) is a rare biliary malformation characterized by congenital dilation of the intrahepatic or extrahepatic bile ducts, frequently accompanied by an anomalous union of the pancreaticobiliary duct. Its incidence is notably higher in Asian populations than in Western populations (1). Without timely intervention, CC may lead to complications such as jaundice, intra-cystic calculi, cholecystitis, pancreatitis, liver abscess, cirrhosis, and malignant transformation (2). Therefore, early diagnosis and surgical intervention are crucial for improving patient outcomes. Currently, radical cyst excision combined with Roux-en-Y hepaticojejunostomy has become the standard surgical treatment for CC, aiming to completely resect the diseased bile duct, reconstruct biliary drainage, and eliminate the risk of malignancy (3).

In recent years, with the rapid advancement of minimally invasive surgical techniques, surgical approaches have evolved from traditional open surgery(OS) to laparoscopic surgery (LS) and, more recently, to robot-assisted surgery (RS) (4). Laparoscopic surgery has become the routine choice in many centers due to its advantages including minimal trauma, faster recovery, and shorter hospital stay. However, its application in children presents certain challenges, such as limited working space, risk of injury to vital structures, and reduced tolerance to prolonged pneumoperitoneum. Moreover, complex laparoscopic procedures may entail inherent drawbacks including loss of tactile feedback, reliance on two-dimensional imaging, and restricted instrument maneuverability, all of which may compromise surgical precision—particularly for surgeons with limited experience (5).

The advent of robotic surgical systems has offered new possibilities to overcome these limitations. Since the first reported case in 2006 (6), robot-assisted surgery—with its three-dimensional high-definition visualization, tremor filtration, and highly articulated wristed instruments—has demonstrated unique potential in pediatric CC surgeries requiring fine dissection and complex reconstruction (7). Theoretically, these technological advantages may translate into more precise cyst excision, more reliable biliary-enteric anastomosis, and fewer postoperative complications.

Although robot-assisted surgery has been widely adopted across various adult surgical specialties (8–10), its application in the pediatric population—particularly for complex procedures such as CC excision—remains fraught with controversy and unresolved issues (7). Currently, studies comparing RS and LS for pediatric CC are predominantly single-center, small-sample retrospective cohort studies, yielding inconsistent conclusions (11–13). For instance, while some studies suggest that RS offers advantages in reducing blood loss and accelerating postoperative recovery, others highlight longer operative times and significantly increased medical costs associated with RS. This inconsistency in evidence, compounded by technical variations across different generations of robotic systems (e.g., da Vinci Si, Xi, SP), the influence of the surgeon's learning curve, and heterogeneity in patient age and pathological characteristics, complicates comprehensive assessment of the true efficacy and safety of RS. Although relevant meta-analyses have been published (5, 14), comprehensive analyses specifically focusing on a head-to-head comparison between these two minimally invasive modalities remained insufficient—particularly regarding differences in key postoperative complications such as biliary stones, bile leakage, and anastomotic stricture, for which no clear consensus has been established. Moreover, the past two years, particularly 2025, have witnessed a substantial increase in newly published studies (4, 11, 19–20, 21–25). Hence, there was an urgent need to systematically review and update the current body of clinical evidence.

Accordingly, this study aimed to conduct a systematic review and meta-analysis to comprehensively compare perioperative outcomes between RS and LS in the treatment of pediatric CC. By synthesizing the best available evidence, we hoped to further delineate the strengths and limitations of these two minimally invasive approaches, thereby providing evidence-based guidance to assist clinical surgeons in selecting the most appropriate surgical strategy.

## Materials and methods

### Literature search and inclusion criteria

This systematic review was conducted in accordance with the Meta-analysis Of Observational Studies in Epidemiology (MOOSE) guidelines and the Preferred Reporting Items for Systematic Reviews and Meta-Analyses (PRISMA) statement (15).

We systematically searched PubMed, Embase, Web of Science, and the Cochrane Library from inception to December 31, 2025. The search strategy employed terms including “CC,” “congenital biliary dilatation,” “laparoscopy,” “robot,” “da Vinci,” and relevant free-text words in the aforementioned databases. No restrictions were placed on study design, and the search was limited to English-language publications.

Inclusion criteria were: (1) studies comparing LS and RS for CC in children; (2) studies providing at least one available outcome for meta-analysis. Duplicate publications, conference abstracts, reviews, and studies without full-text access or extractable data were excluded.

### Data extraction and quality assessment

Initially, (Fei Liu) and (Ke Zhang) independently screened the titles and abstracts of all retrieved studies. Subsequently, these two reviewers independently assessed the full texts of potentially eligible studies. Any discrepancies were resolved through discussion or by consulting a third reviewer (Xiaolong Xie) until consensus was reached.(Fei Liu) and (Ke Zhang) independently extracted all data, and the extracted data were cross-checked to ensure consistency.

For continuous variables not reported as mean and standard deviation, we performed data conversion using the methods described by Wan et al. (16) and Cai et al. (17). The following information was extracted from all included studies: first author, publication year, country, number of patients, etc. The Newcastle-Ottawa Scale (NOS) was used to assess the quality of included studies, with a maximum score of 9. Studies scoring less than 5 were considered low quality and were excluded (18).

### Outcome measures

The following outcomes were extracted or calculated for meta-analysis:

**Intraoperative outcomes:** total operative time, cyst excision time, hepaticojejunostomy time, intraoperative blood loss, and conversion to open surgery.

**Postoperative outcomes:**

**Complications**: biliary stones, bile leakage, anastomotic stricture, cholangitis, residual cyst, incision infection, intestinal obstruction, pancreatitis, pancreatic leakage, and overall complications;

**Recovery indicators**: drainage tube indwelling time, postoperative fasting time, and hospital stay.

### Statistical analysis

Statistical analyses were performed using Stata 18. Heterogeneity was assessed using the *I²* statistic and Q test (*P* < 0.1or *I²* > 50% indicated significant heterogeneity). A random-effects model was applied when heterogeneity was present; otherwise, a fixed-effects model was used to pool the data.

## Results

### Literature screening process and baseline characteristics of included studies

A total of 467 records were retrieved through the database search. After screening, 19 retrospective studies (4, 11–13, 19–33) were included in the meta-analysis. All studies were conducted in Asian countries (12 from China, 4 from Japan, 2 from South Korea, and 1 from India), and all were published after 2019, with a concentration between 2023 and 2025.

**Figure 1:**
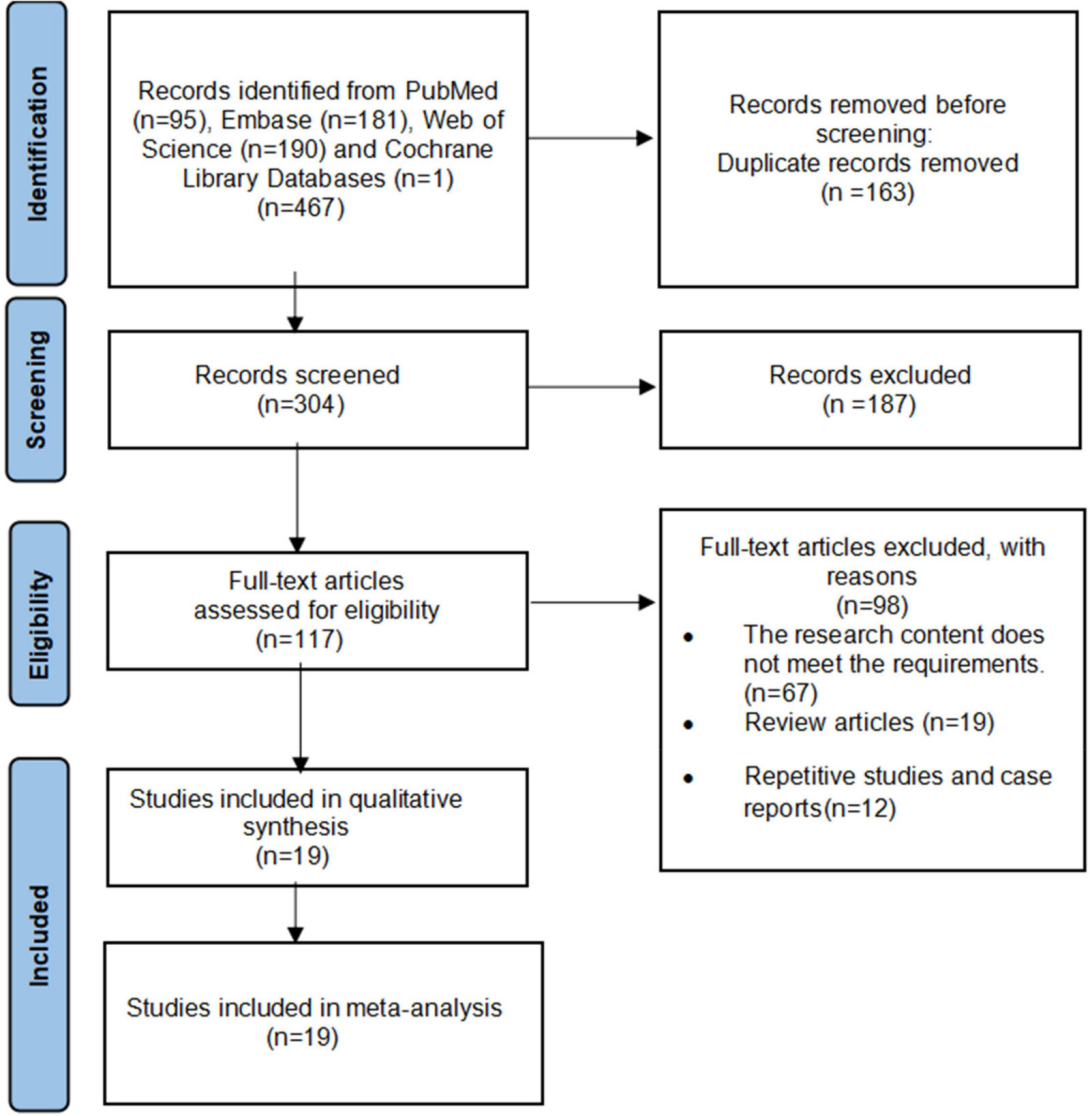
Flowchart of search strategy and study selection.

A total of 2,128 children were included, comprising 888 in the RS group and 1,240 in the LS group. The literature screening process is illustrated in Figure 1. The NOS scores of the 19 studies ranged from 6 to 8 (Table 1), indicating that all included studies were of acceptable quality.

**Table 1:** Characteristics of studies included in meta-analysis.

Author	Year	Country	Research type	Case (N)		Age		Gender		Robot system	NOS
				RS	LS	RS	LS	RS(M/F)	LS(M/F)		
Zhang	2025	China	Retrospective	302	302	13.27–57.6 (m)	13.33–62.07 (m)	84/218	70/232	da Vinci	7
Wang	2025	China	Retrospective	45	72	3.4 (1.2, 5.7) (y)	3.3 (1.0, 5.8) (y)	13/32	25/52	da Vinci Xi	8
Maeda	2025	Japan	Retrospective	29	89	3 (1–6) (y)	2 (1–6) (y)	11/18	20/69	da Vinci Xi	7
Liao	2025	China	Retrospective	32	35	21.17 ± 6.56 (m)	20.60 ± 6.06 (m)	9/23	10/25	da Vinci Xi	8
Liang	2025	China	Retrospective	10	67	2–12 (y)	–	1/9	–	SR-ENS-600	6
Kwon	2025	Korea	Retrospective	63	63	6.0 ± 3.8 (y)	5.7 ± 3.7 (y)	14/49	12/51	da Vinci	7
Kato	2025	Japan	Retrospective	35	92	–	–	–	–	da Vinci	5
Jang	2025	Korea	Retrospective	15	45	87.73 (17.0;124.0) (m)	49.38 (14.0;65.0) (m)	5/10	12/33	da Vinci	6
Chen	2025	China	Retrospective	34	50	76 (46.8–111.8) (d)	62.5 (33.8–95.3) (d)	5/29	5/45	da Vinci	6
Zhang	2024	China	Retrospective	23	26	27 (13,45) (m)	24 (17.3,60.8) (m)	8/15	11/15	da Vinci	7
Yamada	2023	Japan	Retrospective	8	31	6.7 ± 4.7 (y)	6.0 ± 3.8 (y)	–	–	da Vinci	6
Xie	2023	China	Retrospective	8	14	38 (27 ± 49.25) (m)	41 (29 ± 79) (m)	1/7	2/12	da Vinci	6
Pawar	2023	India	Retrospective	20	70	7.7 ± 1.5 (y)	8.6 ± 2.2 (y)	4/16	20/50	da Vinci Si	7
Jin	2023	China	Retrospective	99	34	34 (13–56) (m)	24 (2.875–44.5) (m)	25/74	4/30	da Vinci Xi	7
Lin	2022	China	Retrospective	24	27	30.13 ± 13.88 (m)	33.56 ± 11.56 (m)	9/15	11/16	da Vinci	8
Chen	2022	China	Retrospective	20	22	3. 62 ± 0. 71 (y)	3. 08 ± 0. 82 (y)	6/14	7/15	da Vinci Xi	6
Chi	2021	China	Retrospective	70	70	34.00 ± 27.71 (m)	36.21 ± 32.80 (m)	22/48	22/48	The da Vinci Si	7
Xie	2019	China	Retrospective	41	104	48.00 (30.50–77.50) (m)	28.00 (8.75–53.00) (m)	10/31	25/79	da Vinci	7
Koga	2019	Japan	Retrospective	10	27	5.6 ± 3.4 (y)	5.2 ± 3.8 (y)	–	–	da Vinci	6

m, month; y, year; d, day; “–”: unclear.

Among the 19 studies, Zhang et al. (19) and Kwon et al. (11) employed propensity score matching; Liao et al. (21) and Chen et al. (25) focused on the application of the two surgical approaches in neonates; Liang et al. (22) utilized single-port surgery; Zhang et al. (26) and Lin et al. (31) adopted single-port plus one technique. Regarding robotic platforms, Liang et al. (22) used the novel SR-ENS-600 system, Chi et al. (13) and Pawar et al. (29) employed the da Vinci Si platform, and five studies by Wang et al. (4, 20, 21, 30, 32) utilized the da Vinci Xi platform. The remaining 11 studies (11, 12, 19, 23–28, 31, 33) did not specify the robotic system used.

## Meta-analysis results

### Operative time

**Figure 2:**
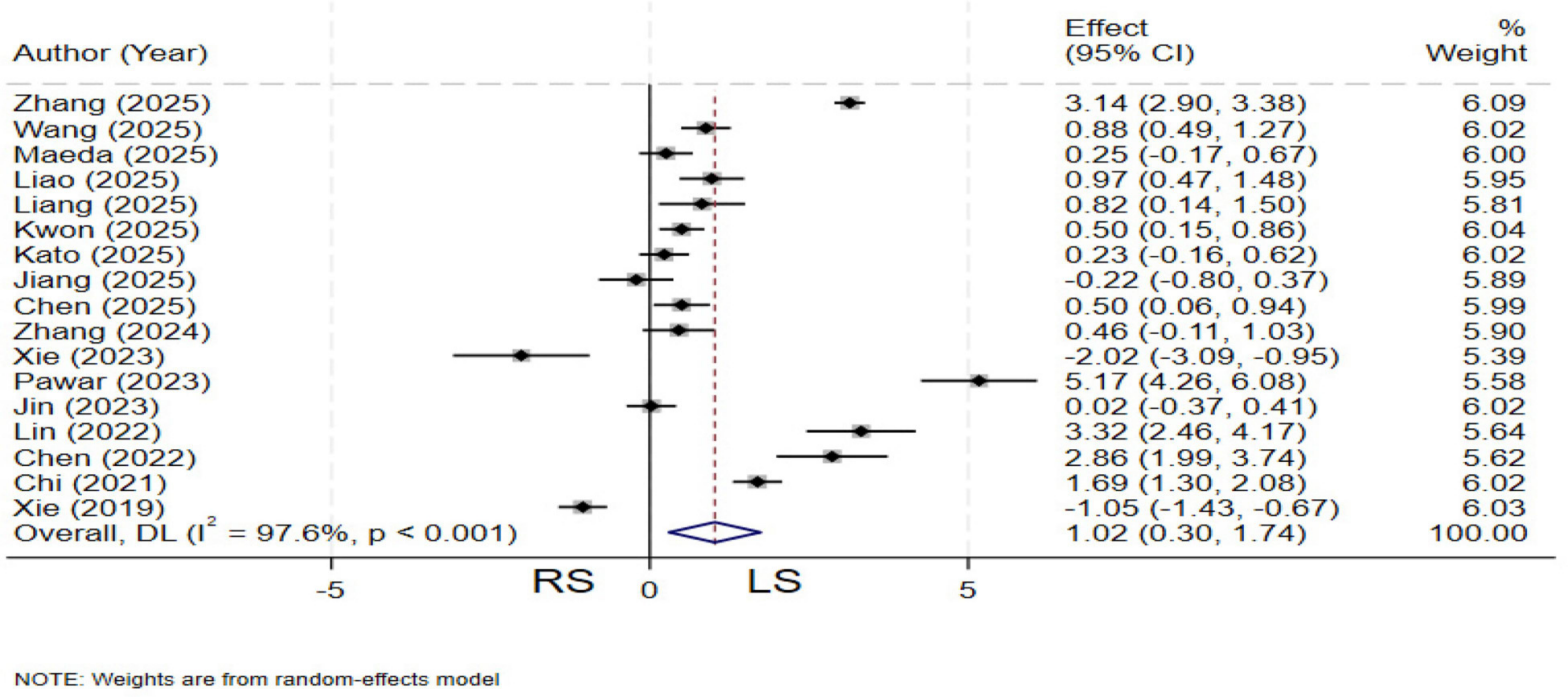
Forest plot of operation time.

A total of 17 studies (4, 11–13, 19–26, 28–32) compared operative time between the two groups. Significant heterogeneity was observed among the studies (*I²* = 97.6%, *P* < 0.001), and a random-effects model was applied. The pooled results demonstrated that the LS group had a significantly shorter operative time compared to the RS group (SMD = 1.02; 95% CI: 0.30–1.74) (Figure 2).

### Intraoperative blood loss

**Figure 3:**
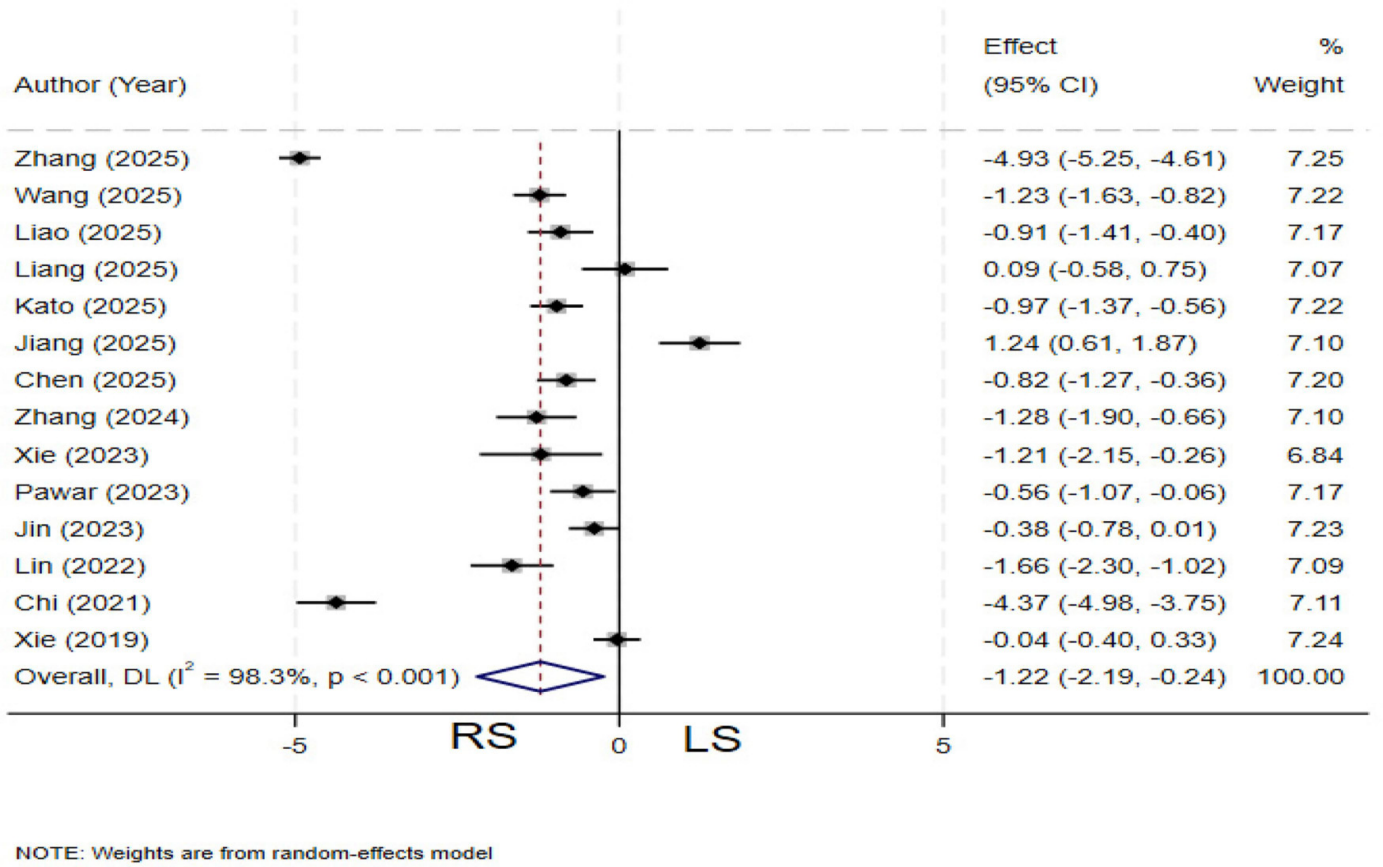
Forest plot of intraoperative blood loss.

A total of 14 studies (12, 13, 19–26, 28–31) compared intraoperative blood loss between the two groups. Significant heterogeneity was observed among the studies (*I²* = 98.3%, *P* < 0.001), and a random-effects model was applied. The pooled results demonstrated that the RS group had significantly less intraoperative blood loss compared to the LS (SMD = −1.22; 95% CI: −2.19∼−0.24) (Figure 3).

### Cyst excision time

**Figure 4:**
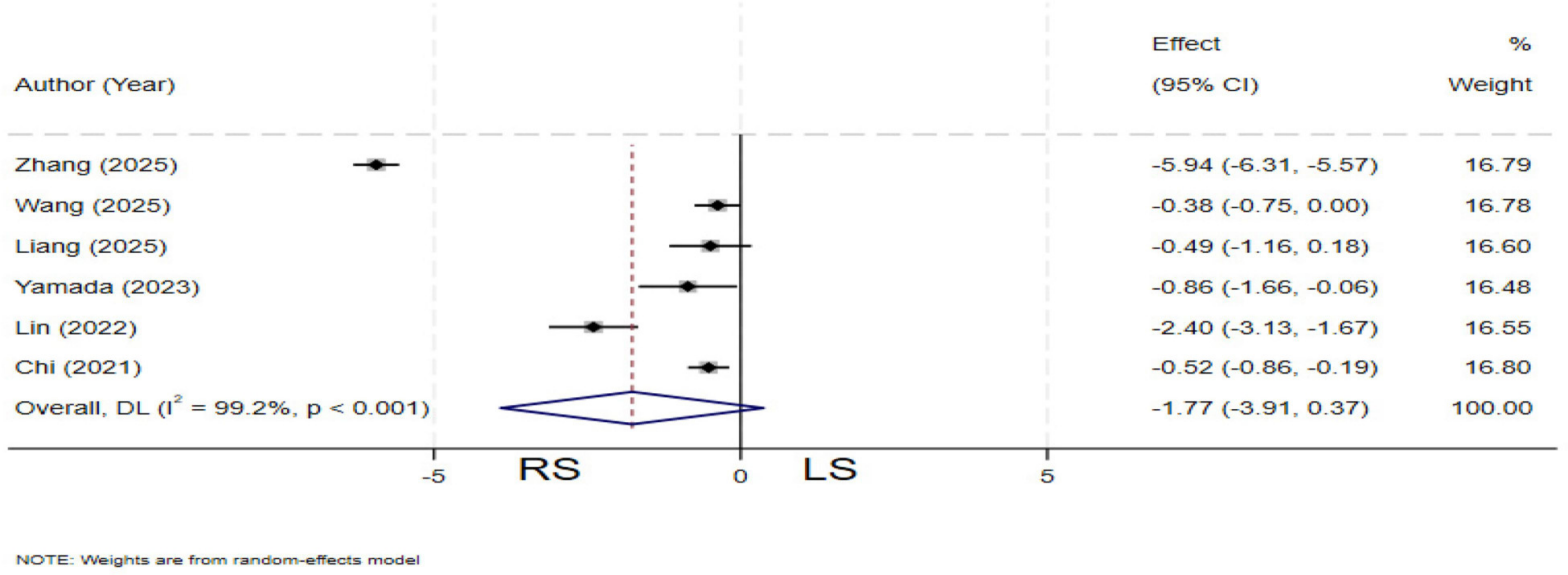
Forest plot of cyst excision time.

Six studies (13, 19, 20, 22, 27, 31) compared cyst excision time between the two groups. Significant heterogeneity was observed among the studies (*I²* = 99.2%, *P* < 0.001), and a random-effects model was applied. The pooled results showed no statistically significant difference in cyst excision time between the RS and LS groups (SMD = −1.77; 95% CI: −3.91∼ 0.37) (Figure 4).

### Hepaticojejunostomy time

**Figure 5:**
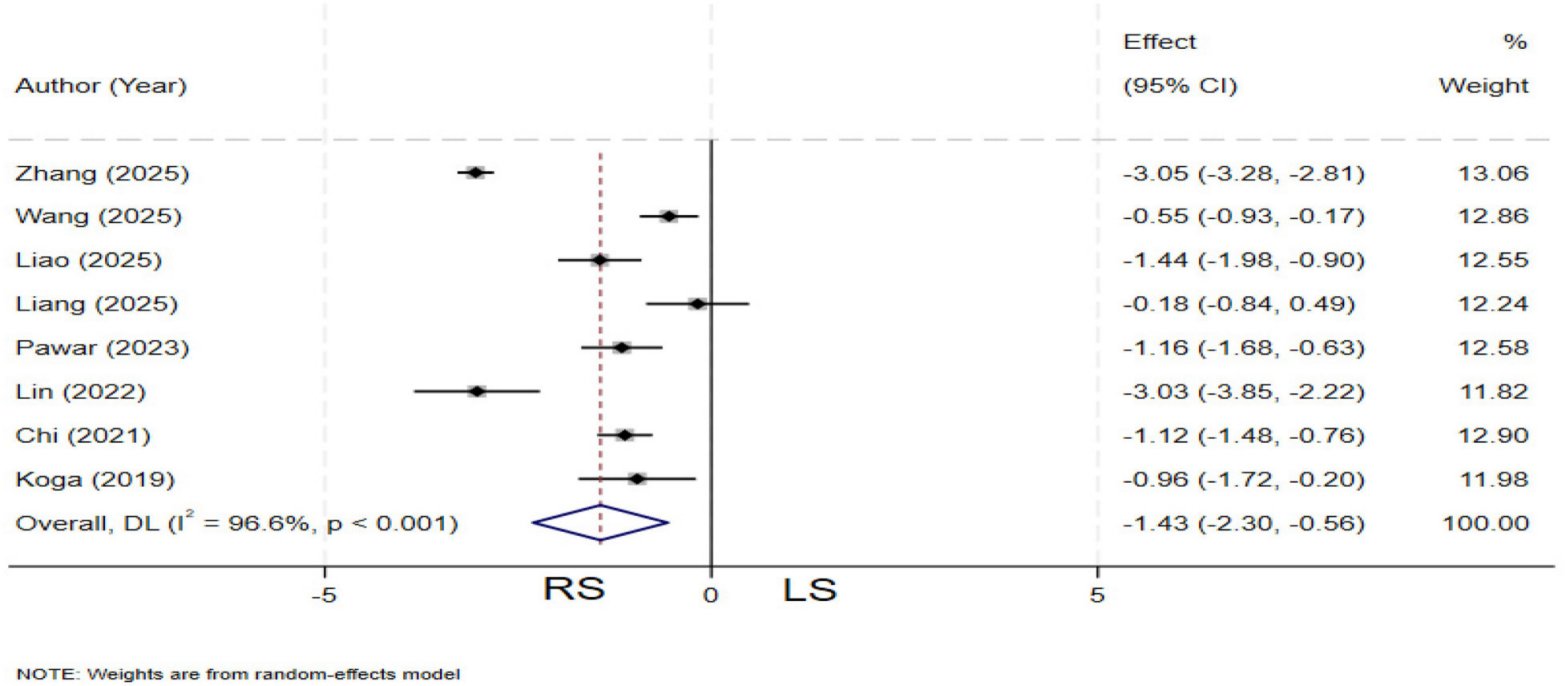
Forest plot of hepaticojejunostomy time.

Eight studies (13, 19–22, 29, 31, 33) compared hepaticojejunostomy time between the two groups. Significant heterogeneity was observed among the studies (*I²* = 96.6%, *P* < 0.001), and a random-effects model was applied. The pooled results demonstrated that the RS group had a significantly shorter hepaticojejunostomy time compared to the LS group (SMD = −1.43; 95% CI: −2.30∼−0.56) (Figure 5).

### Postoperative fasting time

**Figure 6:**
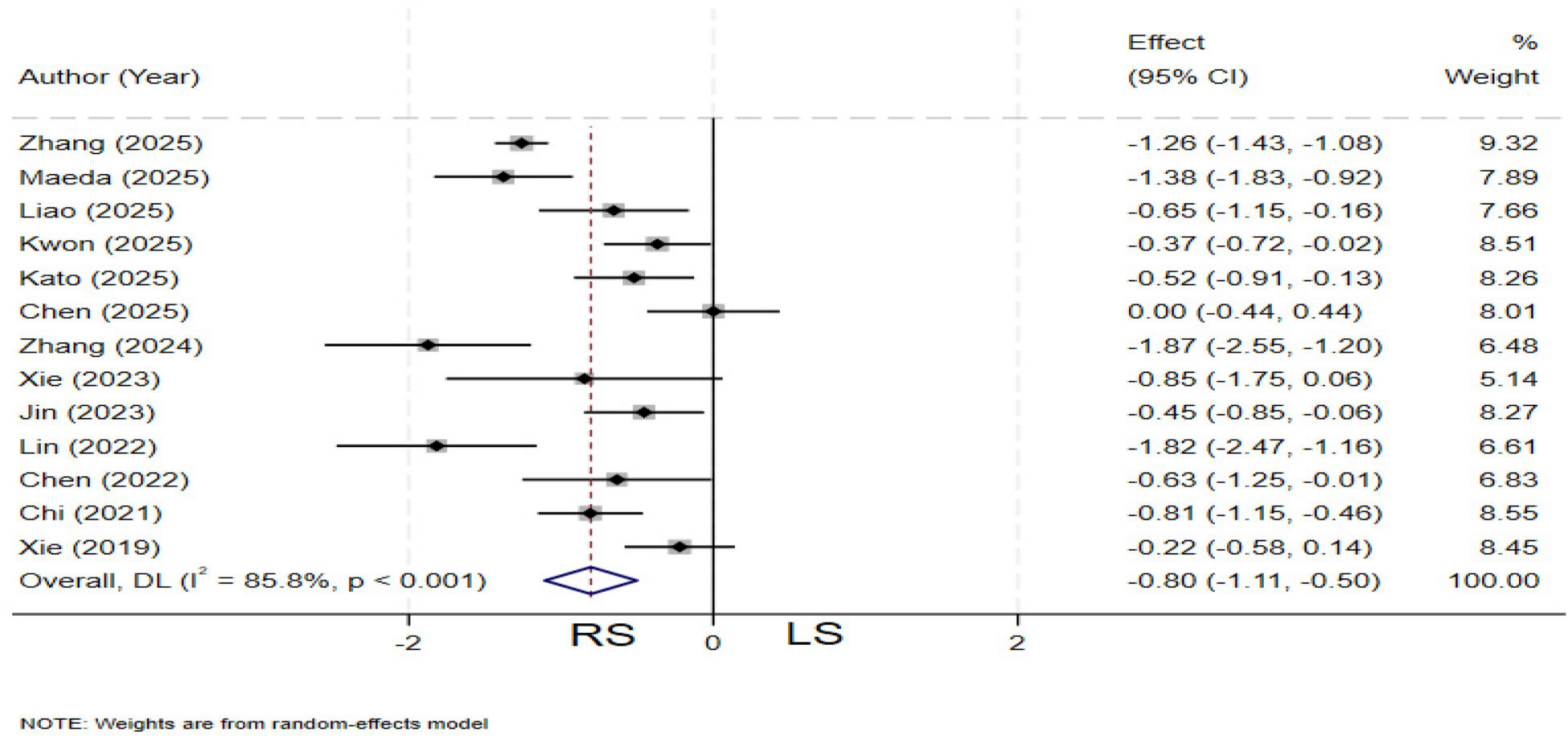
Forest plot of postoperative fasting time.

Thirteen studies (4, 11–13, 19, 21, 23, 25, 26, 28, 30–32) compared postoperative fasting time between the two groups. Significant heterogeneity was observed among the studies (*I²* = 85.8%, *P* < 0.001), and a random-effects model was applied. The pooled results demonstrated that the RS group had a significantly shorter postoperative fasting time compared to the LS group (SMD = −0.80; 95% CI: −1.11∼ −0.50) (Figure 6).

### Drainage tube indwelling time

**Figure 7:**
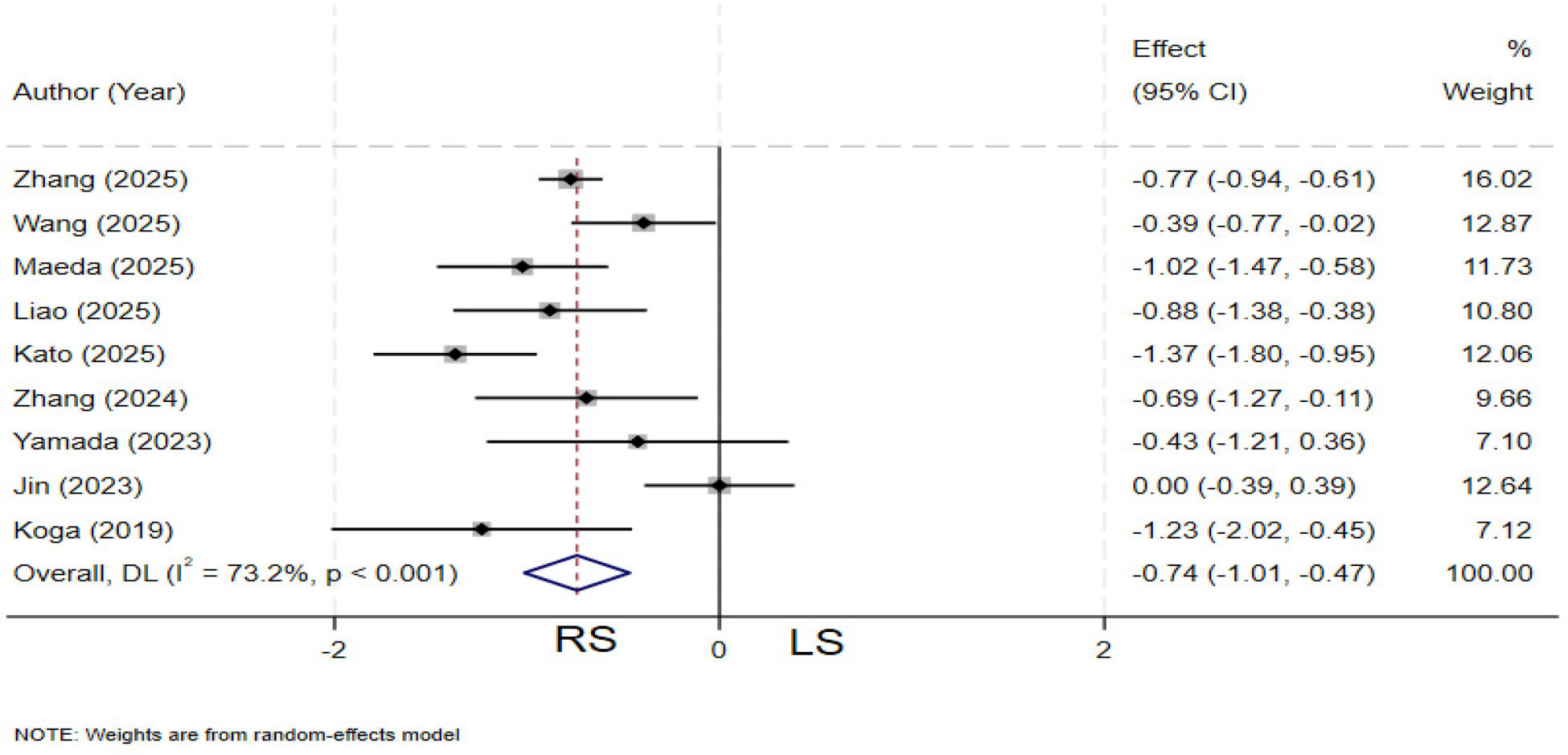
Forest plot of drainage tube indwelling time.

Nine studies (4, 19–21, 23, 26, 27, 30, 33) compared drainage tube indwelling time between the two groups. Significant heterogeneity was observed among the studies (*I²* = 73.2%, *P* < 0.001), and a random-effects model was applied. The pooled results demonstrated that the RS group had a significantly shorter postoperative drainage tube indwelling time compared to the LS group (SMD = −0.74; 95% CI: −1.01∼−0.47) (Figure 7).

### Hospital stay

**Figure 8:**
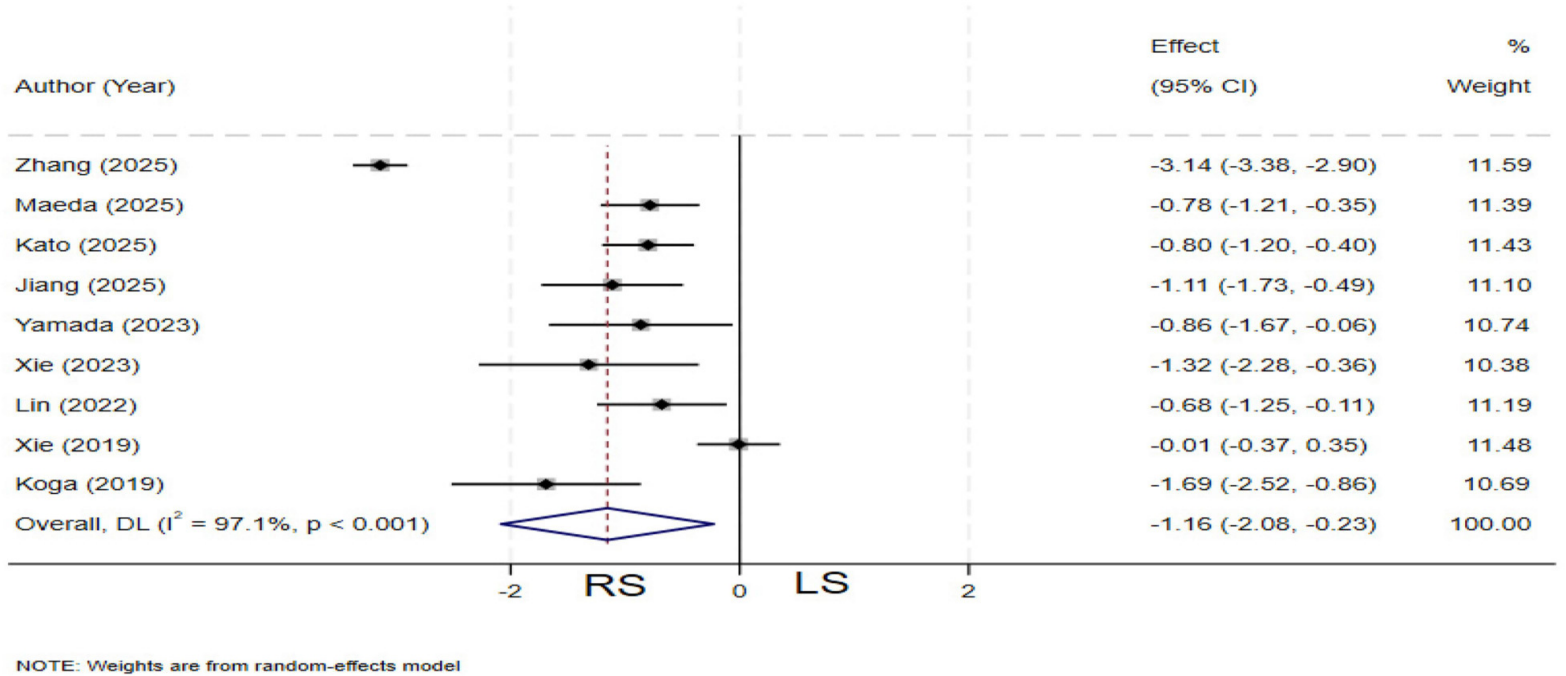
Forest plot of hospital stay.

Nine studies (4, 12, 19, 23, 24, 27, 28, 31, 33) compared hospital stay between the two groups. Significant heterogeneity was observed among the studies (*I²* = 97.1%, *P* < 0.001), and a random-effects model was applied. The pooled results demonstrated that the RS group had a significantly shorter total hospital stay compared to theLS group (SMD = −1.16; 95% CI: −2.08∼−0.23) (Figure 8).

### Conversion

**Figure 9:**
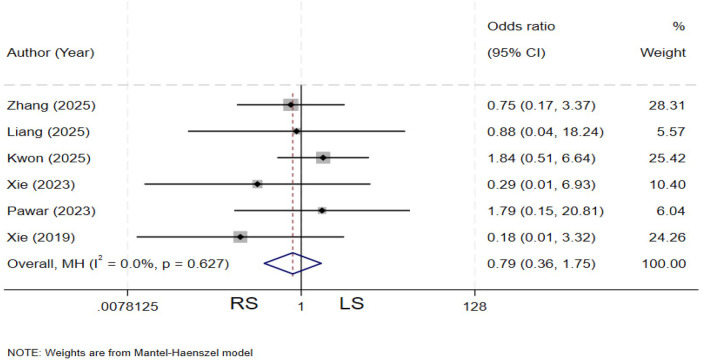
Forest plot of conversion.

Six studies (11, 12, 19, 22, 28, 29) compared the rate of conversion between the two groups. There is no heterogeneity among the various studies (*I²* = 0%, *P* = 0.627). The pooled results showed no statistically significant difference in conversion rate between the two groups (OR = 0.79, 95% CI: 0.36–1.75) (Figure 9).

### Incision infection

**Figure 10:**
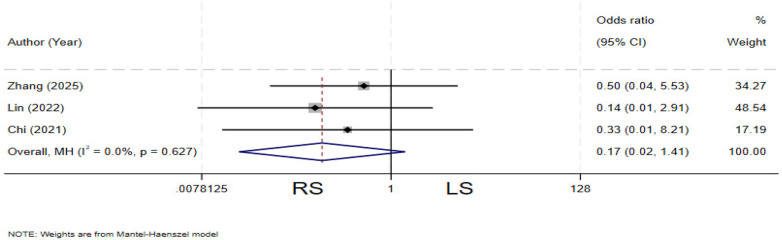
Forest plot of incision infection.

Three studies (13, 19, 31) compared the incidence of incision infection between the two groups. There is no heterogeneity among the various studies (*I²* = 0%, *P* = 0.627). The pooled results showed no statistically significant difference in incision infection rate between the two groups (OR = 0.17, 95% CI: 0.02–1.41) (Figure 10).

### Bile leakage

**Figure 11:**
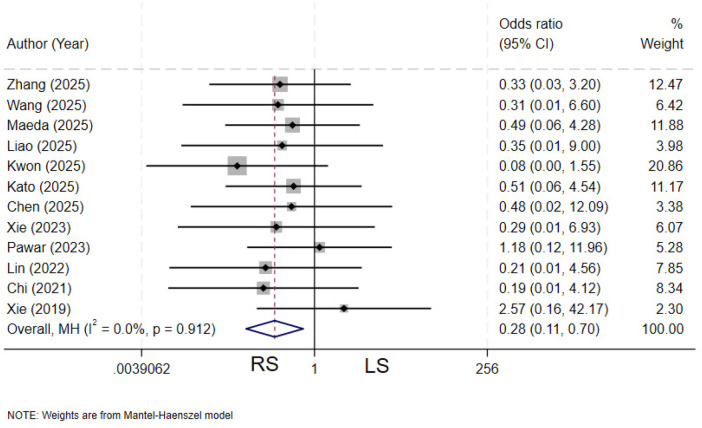
Forest plot of bile leakage.

Twelve studies (4, 11–13, 19–21, 23, 25, 28, 29, 31) compared the incidence of bile leakage between the two groups. There was no heterogeneity among the various studies (*I²* = 0%, *P* = 0.912). The pooled results demonstrated that the RS group had a significantly lower incidence of postoperative bile leakage compared to the LS group (OR = 0.28, 95% CI: 0.11–0.70) (Figure 11).

### Postoperative pancreatitis

**Figure 12:**
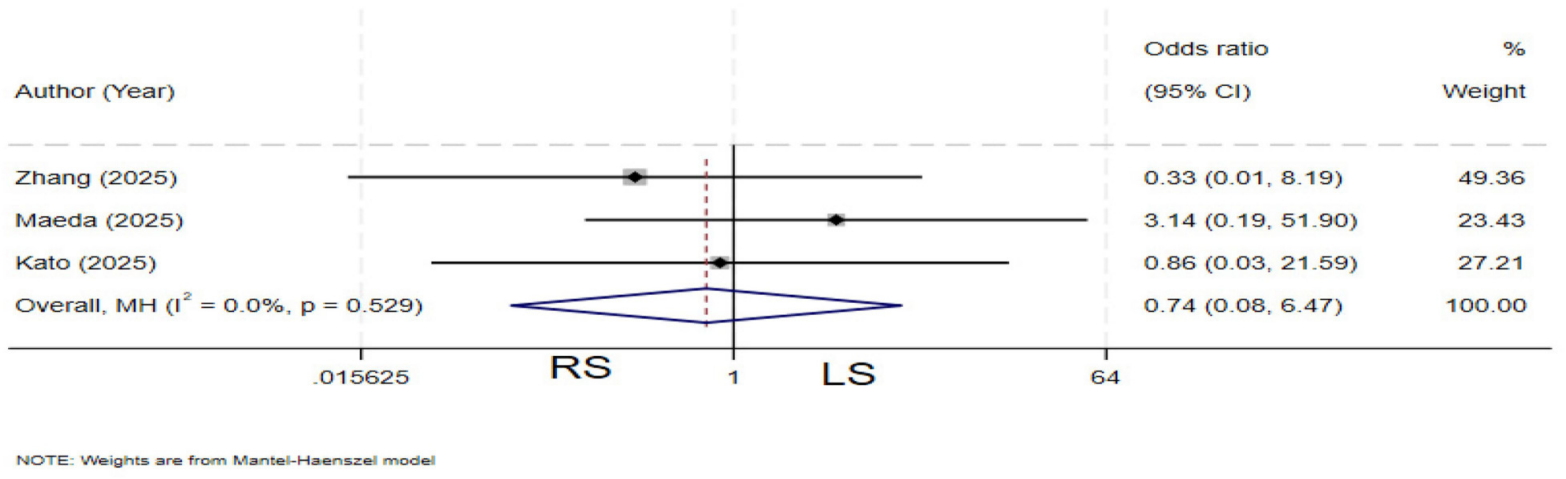
Forest plot of bile leakage.

Three studies (4, 19, 23) compared the incidence of postoperative pancreatitis between the two groups. There is no heterogeneity among the various studies (*I²* = 0%, *P* = 0.529). The pooled results showed no statistically significant difference in the rate of postoperative pancreatitis between the two groups (OR = 0.74, 95% CI: 0.08–6.47) (Figure 12).

### Anastomotic stricture

**Figure 13:**
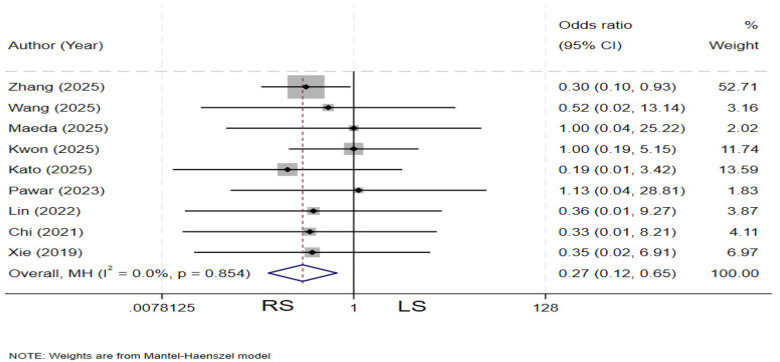
Forest plot of anastomotic stricture.

Nine studies (4, 11–13, 19, 20, 23, 29, 31) compared the incidence of anastomotic stricture between the two groups. There is no heterogeneity among the various studies (*I²* = 0%, *P* = 0.854). The pooled results demonstrated that the RS group had a significantly lower incidence of postoperative anastomotic stricture compared to the LS group (OR = 0.27, 95% CI: 0.12–0.65) (Figure 13).

### Residual cyst

**Figure 14:**
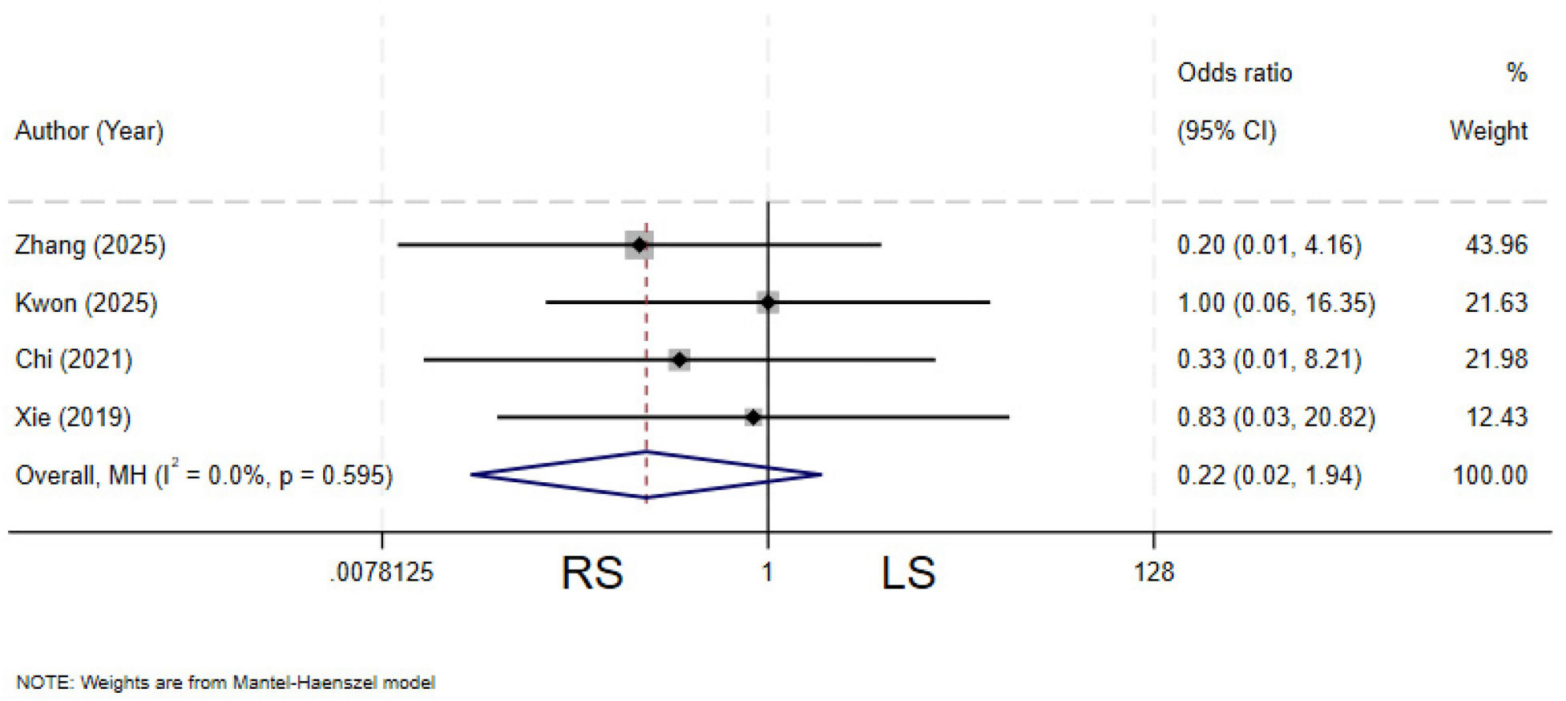
Forest plot of residual cyst.

Four studies (11–13, 19) compared the incidence of residual cyst between the two groups. There is no heterogeneity among the various studies (*I²* = 0%, *P* = 0.595). The pooled results showed no statistically significant difference in the rate of residual cyst between the two groups (OR = 0.22, 95% CI: 0.02–1.94) (Figure 14).

### Biliary stones

**Figure 15:**
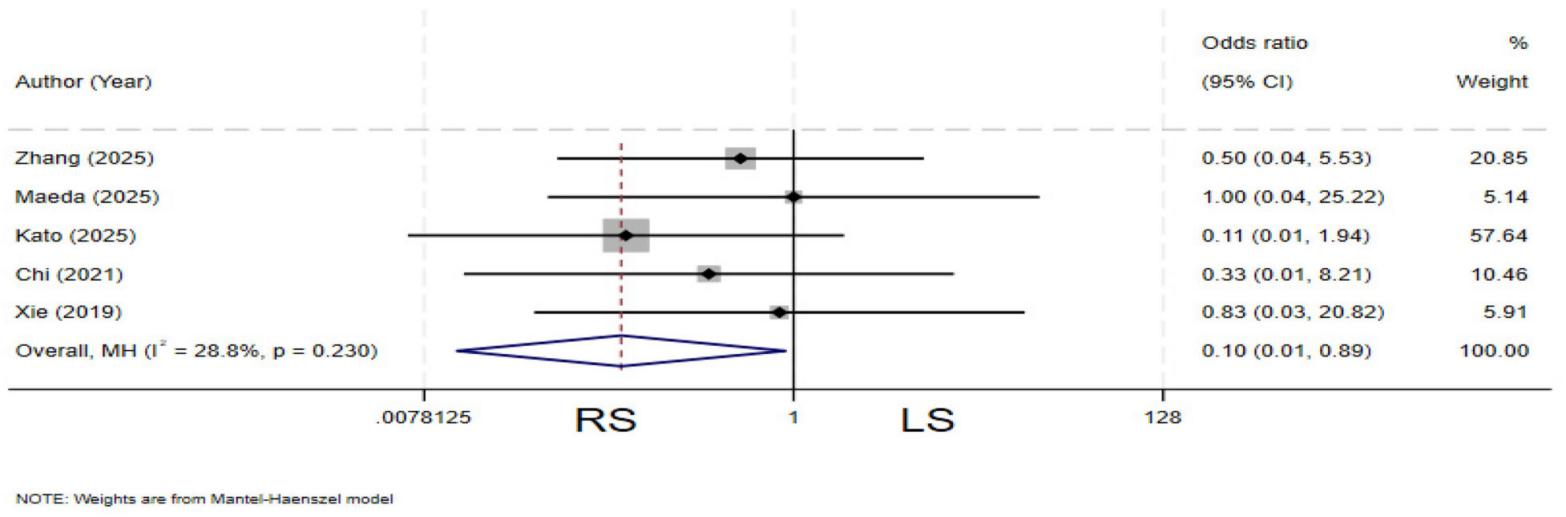
Forest plot of biliary stones.

Five studies (4, 12, 13, 19, 23) compared the incidence of postoperative biliary stones between the two groups. There is no heterogeneity among the various studies (*I²* = 28.8%, *P* = 0.230).The pooled results demonstrated that the RS group had a significantly lower incidence of postoperative biliary stones compared to the LS group (OR = 0.10, 95% CI: 0.01–0.89) (Figure 15).

### Intestinal obstruction

**Figure 16:**
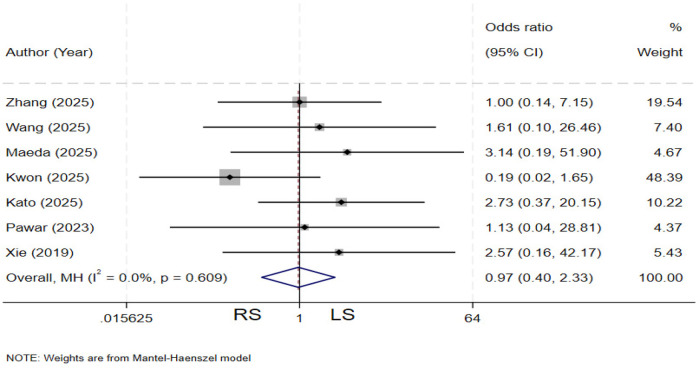
Forest plot of intestinal obstruction.

Seven studies (4, 11, 12, 19, 20, 23, 29) compared the incidence of intestinal obstruction between the two groups. There is no heterogeneity among the various studies (*I²* = 0%, *P* = 0.609). The pooled results showed no statistically significant difference in the rate of intestinal obstruction between the two groups (OR = 0.97, 95% CI: 0.40–2.33) (Figure 16).

### Pancreatic leakage

**Figure 17:**
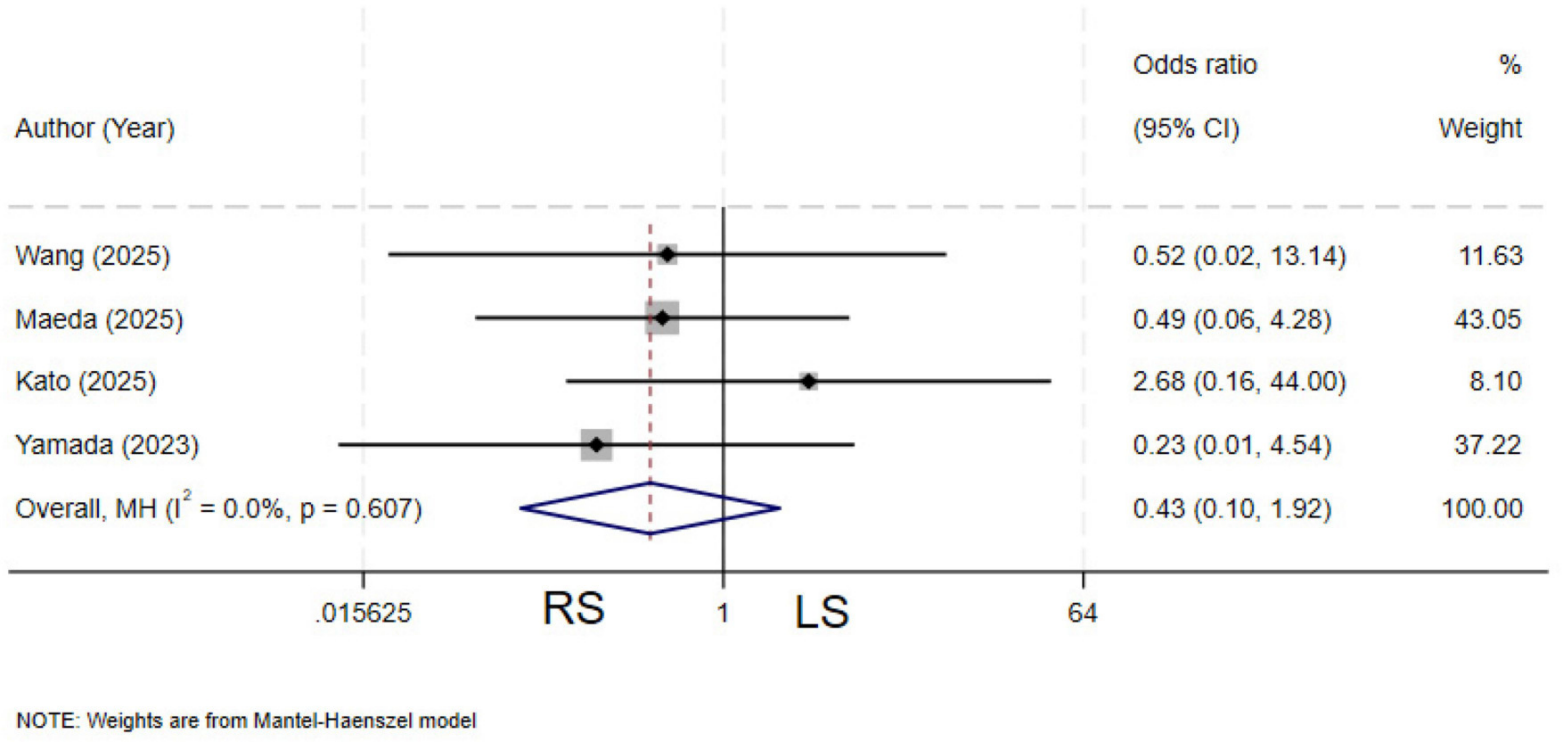
Forest plot of pancreatic leakage.

Four studies (4, 11, 20, 27) compared the incidence of postoperative pancreatic leakage between the two groups. There is no heterogeneity among the various studies (*I²* = 0%, *P* = 0.607).The pooled results showed no statistically significant difference in the rate of pancreatic leakage between the two groups (OR = 0.43, 95% CI: 0.10–1.92) (Figure 17).

### Cholangitis

**Figure 18:**
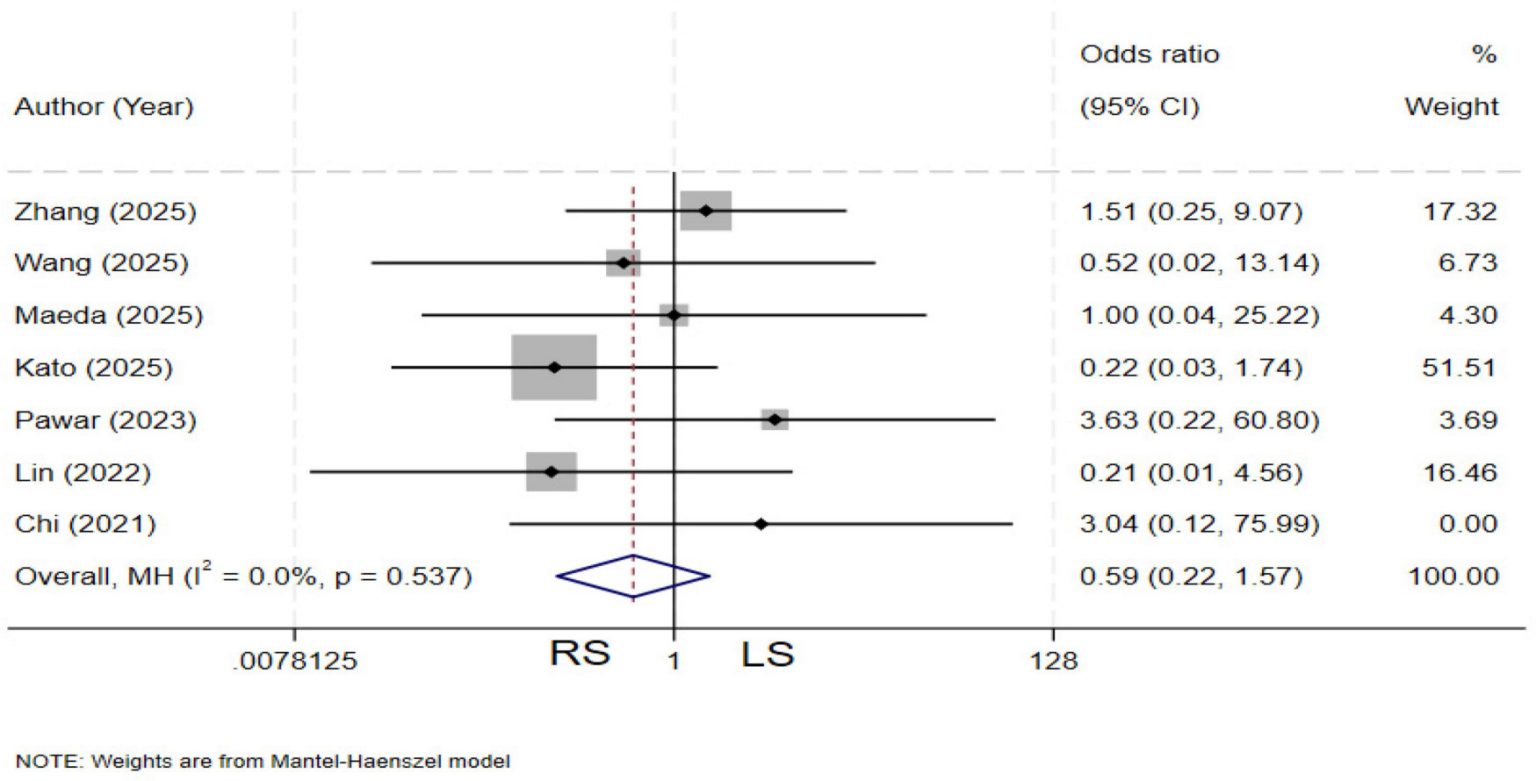
Forest plot of cholangitis.

Seven studies (4, 13, 19, 20, 23, 29, 31) compared the incidence of postoperative cholangitis between the two groups. There is no heterogeneity among the various studies (*I²* = 0%, *P* = 0.537). The pooled results showed no statistically significant difference in the rate of postoperative cholangitis between the two groups. (OR = 0.59, 95% CI: 0.22–1.57) (Figure 18).

### Overall complications

**Figure 19:**
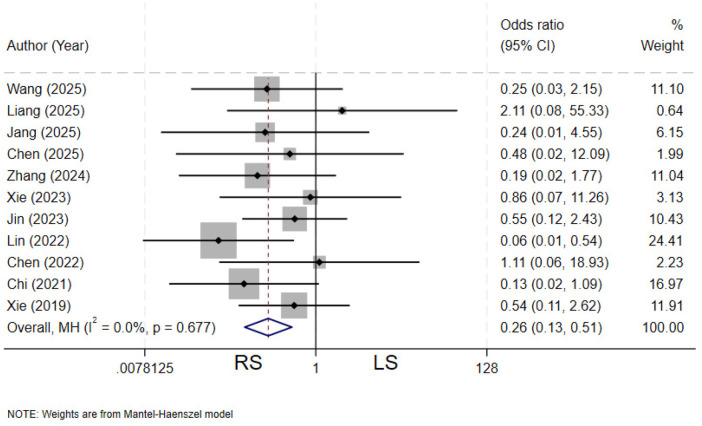
Forest plot of overall complications.

Eleven studies (12, 13, 20, 22, 24–26, 28, 30–32) compared the incidence of postoperative complications between the two groups. There is no heterogeneity among the various studies (*I²* = 0%, *P* = 0.677). The pooled results demonstrated that the RS group had a significantly lower incidence of postoperative complications compared to LS group (OR = 0.26, 95% CI: 0.13–0.51) (Figure 19).

### Subgroup analysis

Based on relevant factors in the included studies, subgroup analyses were performed for four outcomes with significant heterogeneity—operative time, intraoperative blood loss, cyst excision time, and hepaticojejunostomy time—stratified by publication year and robotic platform (Supplementary Figures S1, S2). Subgroup analyses for all four outcomes consistently demonstrated persistent heterogeneity, suggesting that surgeon-related technical factors and the type of robotic platform may represent important sources of heterogeneity.

### Publication bias

Egger's test was conducted to assess publication bias. The results were as follows: conversion (*P* = 0.179), incision infection (*P* = 0.529), bile leakage (*P* = 0.272), pancreatitis (*P* = 0.28), anastomotic stricture (*P* = 0.59), residual cyst (*P* = 0.613), biliary stones (*P* = 0.743), operative time (*P* = 0.485), intraoperative blood loss (*P* = 0.322), cyst excision time (*P* = 0.84), hepaticojejunostomy time (*P* = 0.165), postoperative fasting time (*P* = 0.457), drainage tube indwelling time (*P* = 0.96), hospital stay (*P* = 0.163), Intestinal obstruction (*P* = 0.591), overall complications (*P* = 0.785), pancreatic leakage (*P* = 0.971), and cholangitis (*P* = 0.893). These findings indicated no significant publication bias.

## Discussion

This meta-analysis of 19 retrospective studies systematically compared the efficacy of RS and LS for the treatment of CC in children. All included studies originated from Asian countries, reflecting the relatively higher disease burden of this condition in Asia (1). The pooled results demonstrated that RS was associated with significant advantages in reducing postoperative complication risks and promoting early postoperative recovery.

Intraoperative blood loss was significantly lower in the RS group, a finding that contrasts with some earlier studies (14) but aligns with the majority of recent reports (4, 19–21). This improvement may be attributed to technological advancements and the evolution of robotic platforms. The seven degrees of freedom and tremor filtration of robotic instruments enable precise tissue dissection and hemostasis within the confined pediatric abdominal cavity—particularly in the highly vascularized hilar region—thereby minimizing unnecessary tissue injury and blood loss (4, 34) More importantly, RS was associated with significantly lower incidences of biliary stones, bile leakage, and anastomotic stricture compared to LS, which differs from previous findings (35). The robotic system's articulated instruments, capable of 360-degree rotation, combined with high-definition three-dimensional visualization, facilitate clear identification of complex anatomical structures and precise mucosal apposition during hepaticojejunostomy, thereby enhancing surgical accuracy (22).

Furthermore, the RS group demonstrated superior outcomes in terms of time to first flatus, drainage tube indwelling time, postoperative fasting time, and hospital stay. This may be attributable to the reduced collateral tissue trauma afforded by the robotic system during fine dissection and anastomosis, leading to attenuated local and systemic inflammatory responses and favorable modulation of gastrointestinal hormonal milieu, ultimately resulting in accelerated gastrointestinal functional recovery and shortened overall convalescence (36). A recent network meta-analysis also indicated that RS was associated with the shortest hospital stay compared to both OS and LS (37), further supporting the alignment of RS with the principles of enhanced recovery after surgery (ERAS).

Regarding operative time, our analysis confirmed that RS was associated with significantly longer total operative time than LS, consistent with the majority of published literature (5, 14, 37). The extended time was primarily attributable to robotic system docking, setup, and more meticulous intraoperative dissection. Notably, however, despite the longer total operative time, RS required significantly less time for the core reconstructive step—hepaticojejunostomy—while no statistically significant differences were observed in cyst excision time or the incidence of residual cyst. These findings suggest that the technical advantages of robotics are primarily realized during the reconstructive phase rather than the excisional phase, and that once the robotic system is fully deployed, it may confer greater efficiency in critical procedural steps.

Considerable heterogeneity was observed among several continuous outcome variables, including operative time and intraoperative blood loss. Subsequent subgroup analyses stratified by publication year and robotic platform suggested that surgeon experience and technological platform iteration are likely key contributors to this heterogeneity.

First, previous studies have indicated that the learning curve for robotic CC surgery in children plateaus at approximately 14 cases, with more than 20 hepatobiliary surgeries required for full adaptation to the robotic system (38, 39). Early-series studies were often conducted by teams in the early phase of the learning curve, inevitably resulting in longer operative times. Our subgroup analysis revealed a narrowing of the intergroup difference in operative time in more recent publications, likely reflecting the progressive overcoming of the learning curve across different centers. Operative time is highly dependent on surgeon experience, team coordination, and platform performance, and is particularly sensitive to learning curve effects. In contrast, postoperative complications and recovery parameters represent more definitive endpoint outcomes; once the quality of key procedural steps is improved through robotic assistance, the resultant clinical benefits demonstrate greater stability and reproducibility. Thus, we propose that the evaluation of robotic surgery should shift focus from operative time to outcome improvement, as the value of reducing severe complications and accelerating recovery may far outweigh the cost of increased operative time. Nevertheless, recent academic attention has expanded from perioperative safety to the potential long-term neurodevelopmental effects of prolonged anesthesia in infants (40), future studies should incorporate long-term neurodevelopmental follow-up assessments.

Second, robotic surgical systems are undergoing rapid iterative advancement. From the earlier Si platform to the latest Xi or SP systems, significant improvements have been achieved in instrument articulation, image quality, and arm collision avoidance. The observed heterogeneity in outcomes across different robotic platforms in our subgroup analysis likely reflects intergenerational differences in surgical workflow optimization and hilar exposure efficiency. Newer-generation platforms are designed to streamline procedural steps and reduce intraoperative adjustments, directly contributing to shortened operative time.

Notably, the broad age range of included patients and the application of single-port techniques represent two particularly important considerations in this meta-analysis, with direct implications for the interpretation and generalizability of our findings.

First, with respect to patient age, the included studies spanned a wide age range from neonates to adolescents under 18 years. Age is a major determinant of surgical strategy and outcomes in pediatric populations. In neonates and small infants, the extremely limited intra-abdominal space poses substantial challenges for robotic arm positioning and maneuverability (22). Although our meta-analysis revealed no significant difference in cyst excision time between groups, RS may be associated with further prolongation of operative time in younger patients due to spatial constraints.

Second, regarding single-port techniques, several included studies employed transumbilical single-incision robotic surgery. Single-port robotic surgery represents a cutting-edge direction in minimally invasive surgery, with the primary advantage of consolidating multiple trocar sites into a single umbilical incision; it has demonstrated favorable clinical outcomes and excellent cosmetic results (26). However, a limitation of single-port platforms in pediatric surgery is the relatively small abdominal cavity in children, which restricts the range of robotic arm motion and may compromise surgical efficiency—particularly during deep hilar anastomosis (22). Some studies have proposed a single-port-plus-one technique to address the spatial limitations in children and avoid instrument collision inherent to single-incision surgery (26, 31). Nevertheless, given the limited number of relevant studies and small sample sizes, extrapolation of findings from single-port studies to the general pediatric population warrants considerable caution.

### Study limitations and implications

Several limitations of this study must be acknowledged. (1) All 19 included studies were retrospective cohort studies; no randomized controlled trials were available. The selection of surgical approach was predominantly based on surgeon preference, institutional availability, and family choice rather than random assignment, introducing potential selection bias and confounding that could not be fully controlled. (2) For continuous variables not reported as mean and standard deviation in original studies, we employed previously validated methods for data transformation and pooling, which may have introduced some degree of measurement error. Additionally, inter-study variations in complication definitions, specific surgical techniques (e.g., anastomotic methods), and perioperative management protocols may have contributed to heterogeneity. (3) All included studies originated from Asian countries, limiting the generalizability of our findings to non-Asian populations. Moreover, given the rapid evolution of robotic technology, our analysis aggregated studies utilizing different generations of robotic systems and may not fully reflect the performance of the latest platforms. (4) Imbalances across studies in baseline characteristics such as patient age, cyst type (e.g., type I vs. type IV), and preoperative complications (e.g., cholangitis, pancreatitis) could not be fully adjusted for through subgroup analyses, potentially affecting the robustness of our conclusions. (5) We were unable to analyze healthcare costs—a critical outcome measure. The substantial expenditures associated with robotic system acquisition, maintenance, and consumables represent a major barrier to widespread adoption. Future health economic evaluations should integrate short-term recovery benefits, cost savings from reduced complications, and long-term health gains to conduct comprehensive cost-effectiveness analyses.

Despite these limitations, this study—as the largest and most up-to-date comprehensive synthesis of current evidence—offers important insights for clinical practice. (1) Although previous meta-analyses on this topic have been published, they were largely confined to data prior to 2024 and evaluated a narrower range of outcomes. In contrast, the present study provides an updated and more comprehensive evidence summary incorporating the latest studies published up to December 2025. (2) Our findings indicate that in appropriately selected pediatric patients, particularly those requiring precise biliary-enteric anastomosis, RS is a safe and effective option in well-resourced centers, offering potential advantages in terms of reduced complication risks and accelerated postoperative recovery. (3) Through subgroup analyses, we further confirmed that surgeon experience (learning curve) and robotic platform iteration are core moderating variables driving heterogeneity in efficiency outcomes such as operative time. This finding suggests that pooling studies involving operators at different experience levels or utilizing different platform generations may lead to systematic underestimation or overestimation of the intrinsic efficacy of RS. Accordingly, this study provides quantitative evidence to inform institutional decisions regarding technology adoption—when a surgical team is in the early phase of the learning curve, prolonged operative time should not be simplistically attributed to technical inadequacy but rather considered within the broader context of platform usability and training support. Moreover, our results imply that the disadvantage of longer operative time associated with RS may be mitigated through accumulated surgical experience and technological advancements, underscoring the importance of continuous technical refinement and standardized surgical training, while also reinforcing clinician confidence in continuing to pursue this technique.

## Conclusion

This meta-analysis synthesizes current evidence to demonstrate that, in the treatment of pediatric CC, RS offers potential advantages over LS in terms of reducing postoperative biliary-related complications, decreasing intraoperative blood loss, accelerating postoperative gastrointestinal function recovery, and shortening hospital stay. Although RS is associated with longer operative time, this limitation may be offset by accumulated surgical experience and technological platform evolution. Nevertheless, these benefits must be weighed against contextual factors including patient age, robotic platform type, application of single-port techniques, and higher healthcare costs. The current evidence base is primarily derived from retrospective studies conducted in Asian countries and lacks long-term follow-up data. Well-designed multicenter prospective studies or randomized controlled trials are urgently needed to provide higher-level evidence and further validate our conclusions.

## References

[1] Baison GN Bonds MM Helton WS Kozarek RA. Choledochal cysts: similarities and differences between Asian and Western countries. World J Gastroenterol. (2019) 25:3334–43. 10.3748/wjg.v25.i26.3334 31341359 PMC6639560

[2] Brown ZJ Baghdadi A Kamel I Labiner HE Hewitt DB Pawlik TM. Diagnosis and management of choledochal cysts. HPB (Oxford). (2023) 25:14–25. 10.1016/j.hpb.2022.08.010 36257874

[3] Qu X Cui L Xu J. Laparoscopic surgery in the treatment of children with choledochal cyst. Pak J Med Sci. (2019) 35:807–11. 10.12669/pjms.35.3.85 31258599 PMC6572986

[4] Maeda T Uchida H Shirota C Tainaka T Makita S Yasui A Comparison of postoperative outcomes of open, laparoscopic, and robotic surgery for pediatric choledochal cyst excision. J Pediatr Surg. (2025) 60:162642. 10.1016/j.jpedsurg.2025.162642 40889549

[5] Cao C Xu Z Cen L Mai T Huang J Tian C. Evaluating surgical strategies for pediatric congenital choledochal cysts: a multicenter retrospective study and network meta-analysis. Front Pediatr. (2025) 13:1678421. 10.3389/fped.2025.1678421 41080064 PMC12510841

[6] Woo R Le D Albanese CT Kim SS. Robot-assisted laparoscopic resection of a type I choledochal cyst in a child. J Laparoendosc Adv Surg Tech A. (2006) 16:179–83. 10.1089/lap.2006.16.179 16646713

[7] Cundy T Marcus H Clark J Hughes-Hallett A Mayer E Najmaldin A Robot-assisted minimally invasive surgery for pediatric solid tumors: a systematic review of feasibility and current status. Eur J Pediatr Surg. (2014) 24:127–35. 10.1055/s-0033-1347297 23686663

[8] Di Gialleonardo E Bocchino G Capece G Salvini M Barbaliscia M Malerba G Evaluation of the learning curve in robot-assisted knee arthroplasty: a systematic review. J Exp Orthop. (2025) 12:e70292. 10.1002/jeo2.70292 40655252 PMC12255939

[9] Qu J Harling L. Exploring and analyzing learning curves in robotic-assisted thoracoscopic anatomical lung resections: a systematic review and meta-analysis. JTCVS Open. (2025) 27:184–94. 10.1016/j.xjon.2025.07.011 41169311 PMC12570577

[10] Zhu Y Zou J Wang D Shen D Xue X Zhang W Learning curve and perioperative outcomes of robotic-assisted distal pancreatectomy: a single-center study. J Robot Surg. (2025) 19:716. 10.1007/s11701-025-02908-y 41143940 PMC12559141

[11] Kwon H Namgoong JM Kim DY Kim SC. Comparison of robotic versus laparoscopic cyst excision and hepaticojejunostomy for choledochal cyst in children: a propensity score-matched study. Surg Endosc. (2025) 39:2506–11. 10.1007/s00464-025-11594-8 40011262

[12] Xie X Li K Wang J Wang C Xiang B. Comparison of pediatric choledochal cyst excisions with open procedures, laparoscopic procedures and robot-assisted procedures: a retrospective study. Surg Endosc. (2020) 34(7):3223–31. 10.1007/s00464-020-07560-1 32347390

[13] Chi S-q Cao G-q Li S Guo J-l Zhang X Zhou Y Outcomes in robotic versus laparoscopic-assisted choledochal cyst excision and hepaticojejunostomy in children. Surg Endosc. (2021) 35:5009–14. 10.1007/s00464-020-07981-y 32968912

[14] Zhang K Zhao D Xie X Wang W Xiang B. Laparoscopic surgery versus robot-assisted surgery for choledochal cyst excision: a systematic review and meta-analysis. Front Pediatr. (2022) 10:987789. 10.3389/fped.2022.987789 36389347 PMC9643691

[15] Moher D Liberati A Tetzlaff J Altman DG. Preferred reporting items for systematic reviews and meta-analyses: the PRISMA statement. PLoS Med. (2009) 6:e1000097. 10.1371/journal.pmed.1000097 19621072 PMC2707599

[16] Wan X Wang W Liu J Tong T. Estimating the sample mean and standard deviation from the sample size, median, range and/or interquartile range. BMC Med Res Methodol. (2014) 14:135. 10.1186/1471-2288-14-135 25524443 PMC4383202

[17] Cai S Zhou J Pan J. Estimating the sample mean and standard deviation from order statistics and sample size in meta-analysis. Stat Methods Med Res. (2021) 30:2701–19. 10.1177/09622802211047348 34668458

[18] Stang A. Critical evaluation of the Newcastle-Ottawa scale for the assessment of the quality of nonrandomized studies in meta-analyses. Eur J Epidemiol. (2010) 25:603–5. 10.1007/s10654-010-9491-z 20652370

[19] Zhang M-X Tang J-F Cai D-T Li S Li K Chi S-Q Technical performance, surgical workload and patient outcomes of robotic and laparoscopic surgery for pediatric choledochal cyst: a multicenter retrospective cohort and propensity score-matched study. Hepatobiliary Surg Nutr. (2025) 14:726–41. 10.21037/hbsn-24-439 41104222 PMC12521014

[20] Wang H Zheng Z Gan Y Liao X Du Q Liao Y Advantages of the Da Vinci robotic system in choledochal cyst surgery: a multi-dimensional comparative study with traditional laparoscopic techniques. J Robot Surg. (2025) 19:328. 10.1007/s11701-025-02487-y 40576845 PMC12204871

[21] Liao X Zheng Z Wang H Liao Y Du Q Huang L Comparative analysis of Da Vinci robotic surgery and laparoscopic surgery for congenital choledochal cyst in neonates. BMC Pediatr. (2025) 25:489. 10.1186/s12887-025-05811-5 40596919 PMC12219033

[22] Liang J Chang X You F Wang J Qin S Ning Y Pediatric choledochal cysts on a single-incision robotic surgical platform: a non-randomized pilot study. Surg Endosc. (2026) 40:331–41. 10.1007/s00464-025-12242-x 41087574

[23] Kato D Shirota C Uchida H Hinoki A Makita S Ogawa K Safety and efficacy of robot-assisted bile ductoplasty and intrapancreatic bile duct resection in congenital biliary dilatation: a single-center retrospective cohort (2013–2024). J Robot Surg. (2025) 19:618. 10.1007/s11701-025-02782-8 40968256 PMC12446100

[24] Jang J Ko D Youn JK Yang HB Kim HY. Comparison of laparoscopic and robotic surgery of choledochal cyst in pediatrics: single center experience. Surg Endosc. (2026) 40:462–8. 10.1007/s00464-025-12222-1 41131377 PMC12823707

[25] Chen S Gao Z Chen Q. Choledochal cyst in children under six months: is Da Vinci robot-assisted surgery more advantageous? J Laparoendosc Adv Surg Tech A. (2025) 35:252–6. 10.1089/lap.2024.0031 39761018

[26] Zhang L Chen S Lin Y Wang J Qiu X Li L. Comparative study of robotic-assisted single-incision-plus-one port and single-incision laparoscopic choledochal cyst excision. Front Pediatr. (2024) 12:1403358. 10.3389/fped.2024.1403358 39363967 PMC11447616

[27] Yamada S Koga H Seo S Ochi T Shibuya S Yazaki Y Comparison of robotic assistance and laparoscopy for pediatric choledochal cyst: advantages of robotic assistance. Pediatr Surg Int. (2023) 40:1. 10.1007/s00383-023-05588-7 37989795

[28] Xie X Li K Xiang B. Robotic versus laparoscopic surgery for choledochal cyst in children with aberrant hepatic ducts: a retrospective study. Asian J Surg. (2023) 46:4186–90. 10.1016/j.asjsur.2022.11.006 36411170

[29] Pawar J Chinnusamy P Soundappan S Vijai A. Comparison of perioperative surgical outcomes following total robotic and total laparoscopic Roux-en Y hepaticojejunostomy for choledochal cyst in paediatric population: a preliminary report from a tertiary referral centre. Pediatr Surg Int. (2023) 39:139. 10.1007/s00383-023-05414-0 36842154

[30] Jin Y Zhang S Cai D Zhang Y Luo W Chen K Robot-assisted resection of choledochal cyst in children. Front Pediatr. (2023) 11:1162236. 10.3389/fped.2023.1162236 37404555 PMC10315571

[31] Lin S Chen J Tang K He Y Xu X Xu D. Trans-umbilical single-site plus one robotic assisted surgery for choledochal cyst in children, a comparing to laparoscope-assisted procedure. Front Pediatr. (2022) 10:806919. 10.3389/fped.2022.806919 35281244 PMC8914220

[32] Chen S Lin Y Xu D Lin J Zeng Y Li L. Da vinci robotic-assisted treatment of pediatric choledochal cyst. Front Pediatr. (2022) 10:1044309. 10.3389/fped.2022.1044309 36440346 PMC9683341

[33] Koga H Murakami H Ochi T Miyano G Lane GJ Yamataka A. Comparison of robotic versus laparoscopic hepaticojejunostomy for choledochal cyst in children: a first report. Pediatr Surg Int. (2019) 35:1421–5. 10.1007/s00383-019-04565-3 31555861

[34] Delgado-Miguel C Camps J Garavis Montagut I Arredondo-Montero J Hernández-Oliveros F. Robotic-assisted surgery in children under 10 kg: a systematic review. Surg Endosc. (2026) 40:40–58. 10.1007/s00464-025-12429-2 41269313

[35] Zhang MX Chi SQ Cao GQ Tang JF Tang ST. Comparison of efficacy and safety of robotic surgery and laparoscopic surgery for choledochal cyst in children: a systematic review and proportional meta-analysis. Surg Endosc. (2023) 37:31–47. 10.1007/s00464-022-09442-0 35913517

[36] Fleszar MG Zawadzki M Fortuna P Bednarz-Misa I Krauze I Maciejewska K Robot-assisted colorectal cancer surgery mitigates early postoperative immunosuppression and angiogenesis. Int J Mol Sci. (2025) 26:10041. 10.3390/ijms262010041 41155334 PMC12563495

[37] Cui H Wang X An W Chen M Zhang X Shi S. The efficacy and safety of robotic, laparoscopic, and open surgery for pediatric choledochal cysts: a systematic review and network meta-analysis. Transl Pediatr. (2025) 14:1921–31. 10.21037/tp-2025-102 40949915 PMC12433089

[38] Xie X Feng L Li K Wang C Xiang B. Learning curve of robot-assisted choledochal cyst excision in pediatrics: report of 60 cases. Surg Endosc. (2021) 35:2690–7. 10.1007/s00464-020-07695-1 32556766

[39] Liu D Shields M Stricklin C Troxler C Jarc A Feinn R Early learning curve changes in objective performance indicators during robotic cholecystectomy. Front Surg. (2025) 12:1679666. 10.3389/fsurg.2025.1679666 41141693 PMC12550773

[40] Zhou H Sun W Ning L Kang J Jin Y Dong C. Early exposure to general anesthesia may contribute to later attention-deficit/hyperactivity disorder (ADHD): a systematic review and meta-analysis of cohort studies. J Clin Anesth. (2024) 98:111585. 10.1016/j.jclinane.2024.111585 39153353

