# Supplementary material for: Robot-assisted vs. laparoscopic-assisted surgery for choledochal cyst in children: a systematic review and meta-analysis
Source: Front Pediatr. 2026 Apr 28;14:1811576. doi: 10.3389/fped.2026.1811576 (PMC13160894; doi:10.3389/fped.2026.1811576)
Supplement: Supplementary file 2 [file Datasheet2.pdf]

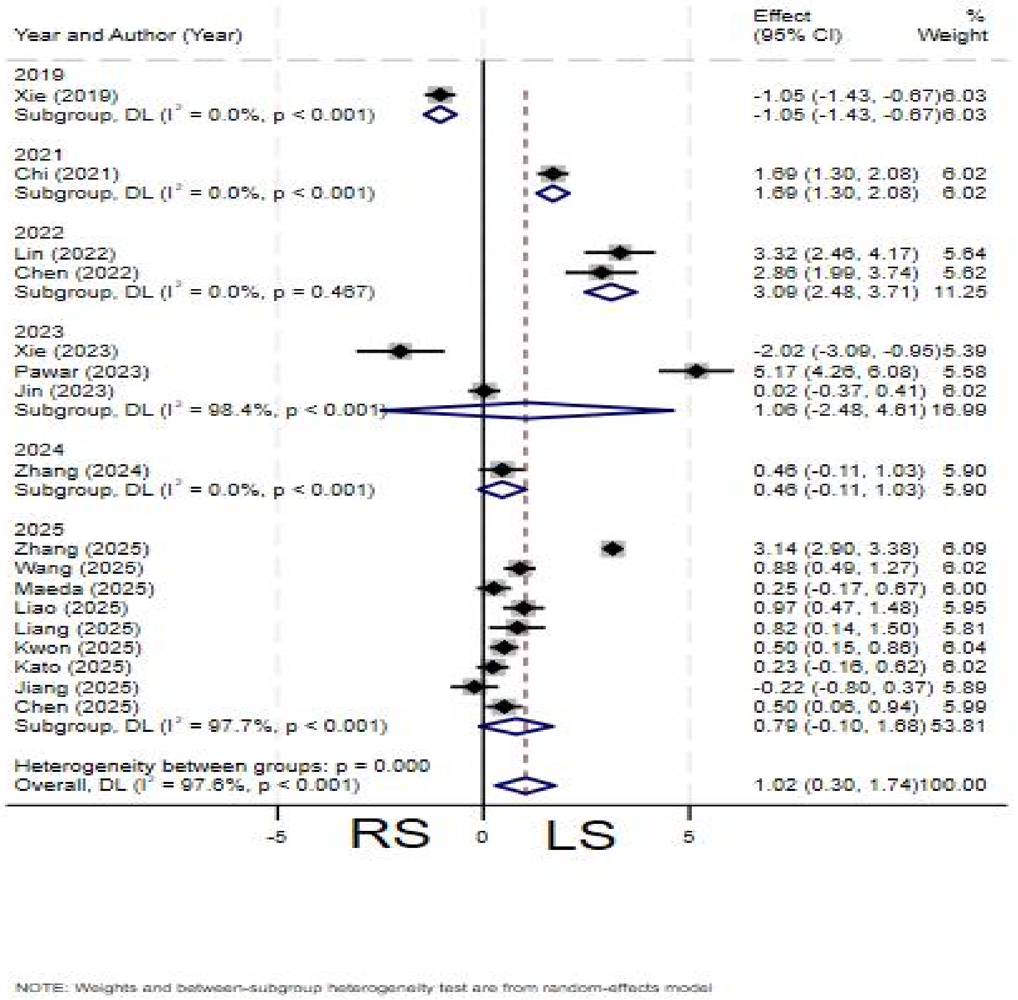

operative time

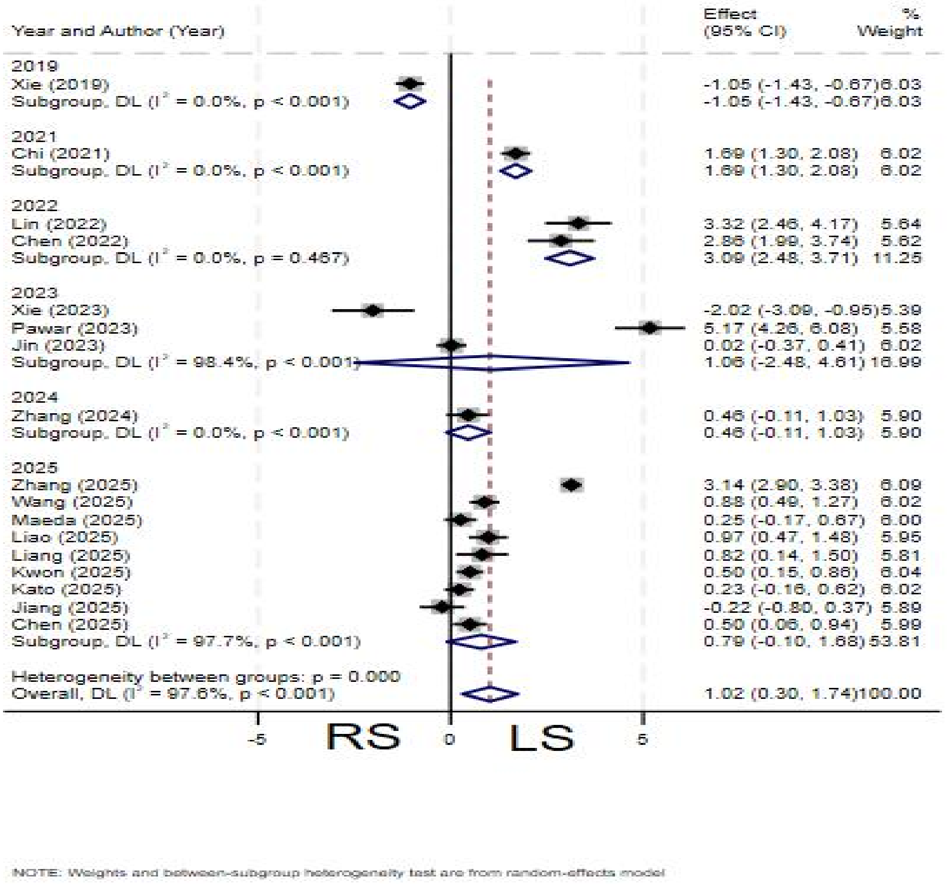

intraoperative blood loss

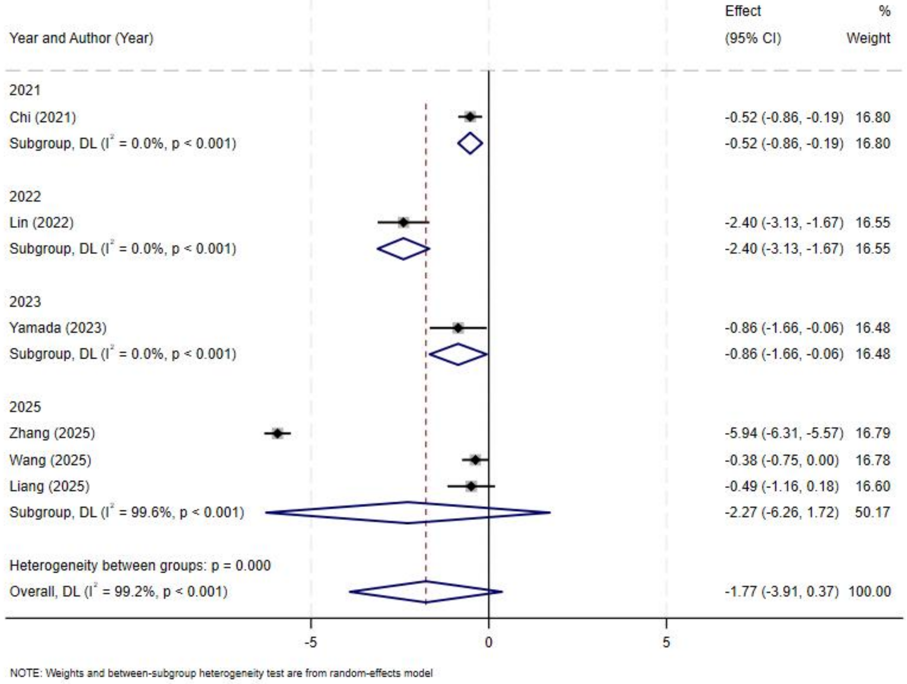

cyst excision time

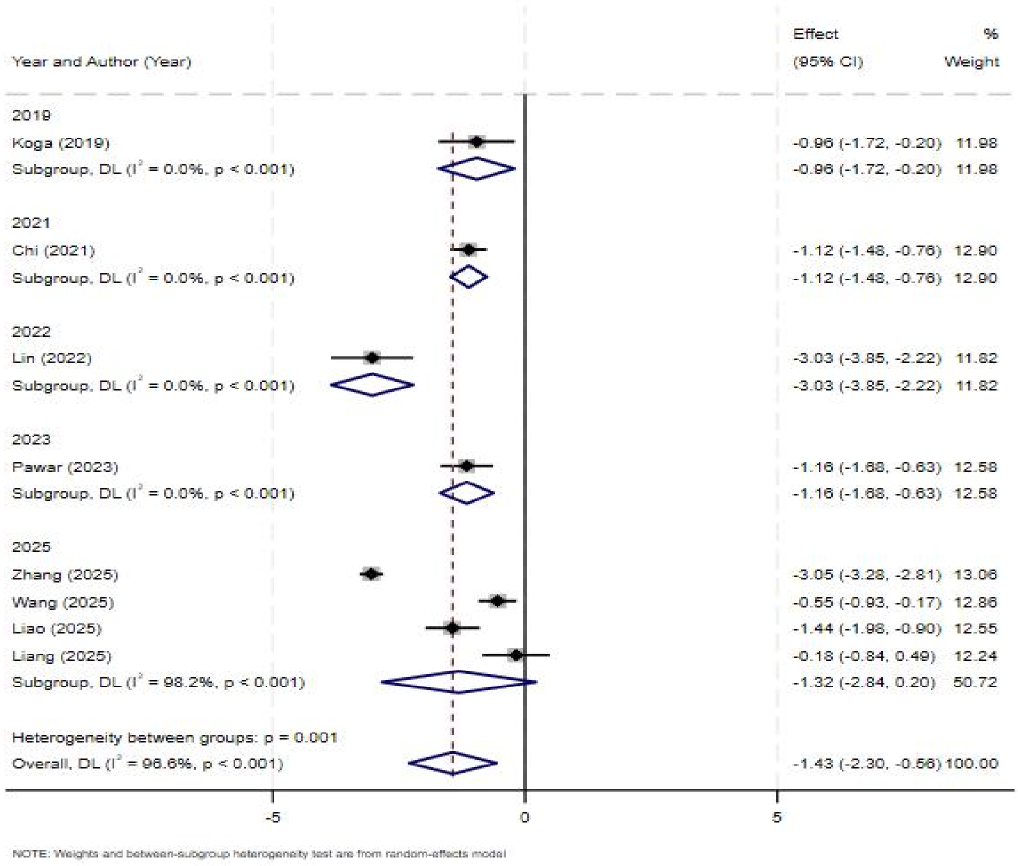

hepaticojejunostomy time
